# Rare Wolbachia genotypes in laboratory Drosophila melanogaster strains

**DOI:** 10.18699/VJGB-22-67

**Published:** 2022-10

**Authors:** A.S. Ryabinin, O.D. Shishkina, Yu.Yu. Ilinsky, R.A. Bykov

**Affiliations:** Institute of Cytology and Genetics of the Siberian Branch of the Russian Academy of Sciences, Novosibirsk, Russia; Institute of Cytology and Genetics of the Siberian Branch of the Russian Academy of Sciences, Novosibirsk, Russia; Institute of Cytology and Genetics of the Siberian Branch of the Russian Academy of Sciences, Novosibirsk, Russia; Institute of Cytology and Genetics of the Siberian Branch of the Russian Academy of Sciences, Novosibirsk, Russia

**Keywords:** Drosophila melanogaster, Wolbachia, genotypes, laboratory stock, Drosophila melanogaster, Wolbachia, генотипы, лабораторный фонд

## Abstract

Symbiotic bacteria of the genus Wolbachia are widespread in Drosophila melanogaster populations. Based on the polymorphism of the Wolbachia genome, the symbionts’ diversity in D. melanogaster is presented by two groups: MEL (wMel, wMel2, wMel3 and wMel4) and CS (wMelCS and wMelCS2). The wMel genotype is predominant in natural D. melanogaster populations and is distributed all over the world. The CS genotypes, on the other hand, are of particular interest because it is unclear how they are maintained in the fruit f ly populations since they should have been eliminated from them due to their low frequency and genetic drift or been replaced by the wMel genotype. However, this is not what is really observed, which means these genotypes are supported by selection. It is known that the wMelPlus strain of the wMelCS genotype can increase the lifespan of infected f lies at high temperatures. The same genotype also increases the intensity of dopamine metabolism in Drosophila compared to the MEL-group genotypes. In the present study, we searched for the rare Wolbachia wMelCS and wMelCS2 genotypes, as well as for new genotypes in wild-type D. melanogaster strains and in several mutant laboratory strains. The symbiont was found in all populations, in 200 out of 385 wild-type strains and in 83 out of 170 mutant strains. Wolbachia diversity in D. melanogaster wild-type strains was represented by the wMel, wMelCS and wMelCS2 genotypes. More than 90 % of the infected strains carried wMel; 9 %, wMelCS2; and only two strains were found to carry wMelCS. No new Wolbachia genotypes were found. The northernmost point reported for the wMelCS2 genotype was Izhevsk city (Udmurtia, Russia). For the f irst time the wMelCS2 genotype was detected in D. melanogaster from the Sakhalin Island, and wMelCS, in the f lies from Nalchik (the North Caucasus). A comparison of Wolbachia genetic diversity between the wild-type laboratory strains and previously obtained data on mutant laboratory strains demonstrated differences in the frequencies of rare CS genotypes, which were more prevalent in mutant strains, apparently due to the breeding history of these Drosophila strains

## Abstract (ru)

Симбиотические бактерии рода Wolbachia широко распространены в популяциях Drosophila melanogaster. На основе полиморфизма перестроек генома разнообразие Wolbachia у D. melanogaster подразделяется на две клады: MEL (генотипы wMel, wMel2, wMel3 и wMel4) и CS (wMelCS и wMelCS2). Генотип wMel доминирует в природных популяциях D. melanogaster и распространен по всему миру. Генотипы CS-клады представляют особый интерес, поскольку неизвестно, как они поддерживаются в популяциях D. melanogaster. При низкой частоте встречаемости они должны элиминироваться вследствие генетического дрейфа или вытесняться генотипом wMel, чего в действительности не происходит. Следовательно, эти генотипы поддерживаются отбором. Например, штамм wMelPlus (генотип wMelCS) способен увеличивать продолжительность жизни мух при повышенных температурах. Генотип wMelCS также увеличивает интенсивность дофаминового метаболизма у дрозофил по сравнению с генотипами MEL-клады. В настоящей работе проведен поиск редких генотипов Wolbachia wMelCS и wMelCS2, а также новых генотипов в линиях D. melanogaster дикого типа и в отдельных мутантных линиях лабораторного фонда. Симбионт был выявлен во всех популяционных выборках у 200 из 385 линий дикого типа и у 83 из 170 мутантных. Разнообразие Wolbachia в линиях D. melanogaster дикого типа представлено генотипами wMel, wMelCS и wMelCS2. Более 90 % инфицированных линий несут Wolbachia wMel генотипа, 9 % – wMelCS2, и только в двух линиях обнаружен wMelCS. Новых генотипов Wolbachia не зафиксировано. Для генотипа wMelCS2 отмечена наиболее северная точка распространения – Ижевск (Уд- муртия). Впервые показано присутствие генотипа wMelCS2 в линии D. melanogaster из популяции о. Сахалин, а в линии из популяции г. Нальчик – генотипа wMelCS. Сравнение генетического разнообразия Wolbachia меж- ду лабораторными линиями дикого типа и ранее полученными данными для мутантных лабораторных линий показало различие в частотах редких генотипов CS-группы, у мутантных линий их больше, что может быть связано с историей поддержания линий Drosophila.

## Introduction

Symbiotic bacteria of the Wolbachia genus are widespread in Drosophila melanogaster populations (Riegler et al., 2005; Richardson et al., 2012; Ilinsky, 2013; Bykov et al., 2019). Apart from a number of point mutations, these Wolbachia genomes differ by a series of the rearrangements that can be easily detected by polymerase chain reaction (PCR) assay followed by electrophoretic analysis as per M. Reigler et al. (2005). Their polymorphism has enabled one to distinguish MEL (wMel, wMel2, wMel3 and wMel4) and CS (wMelCS and wMelCS2) group of genotypes (Riegler et al., 2005; Ilinsky, 2013). The wMel genotype, whose name is similar to that of the strain, prevails in D. melanogaster, the others have either rare or local spread (Riegler et al., 2005; Ilinsky, Zakharov, 2007a, b; Ilinsky, 2013; Bykov et al., 2019), e. g. while being widely spread in the world, the wMelCS genotype is rare, where its prevalence does not exceed 10 % (Riegler et al., 2005; Ilinsky, Zakharov, 2007a, b; Serga et al., 2014; Bykov et al., 2019).

Meanwhile, the wMelCS2 genotype is often detected in the D. melanogaster populations of Eastern Europe, Central and Northern Asia and Western Siberia, whose prevalence in some samples could reach up to 40 % (Riegler et al., 2005; Ilinsky, Zakharov, 2007a, b; Ilinsky, 2013; Bykov et al., 2019). In the strains of South and South-East Asia, singular cases of wMel2 genotype presence have been detected (Riegler et al., 2005; Bykov et al., 2019), while the wMel4 genotype was first registered in the Sinai Peninsula, and no other data are currently available regarding its spread (Ilinsky, 2013). The wMel3 genotype was found only in a single laboratory strain and is most likely absent in the wild (Riegler et al., 2005).

Detailed genome analysis of the Wolbachia bacteria in D. melanogaster confirmed the abovementioned subdivision and enabled one to subdivide the MEL and CS groups into several clades (Richardson et al., 2012; Chrostek et al., 2013; Early, Clark, 2013; Ilinsky, 2013). Thus, the most widespread wMel genotype has four (I, II, III and V) clades, and the wMel2 genotype – two (IV and VIII). As for the CS group, it has only one clade (Richardson et al., 2012; Chrostek et al., 2013; Ilinsky, 2013). Analysis of the nucleotide polymorphism of the full genomes of the wMelCS and wMelCS2 genotypes detected four haplotypes (Bykov et al., 2019). One of which is present in wild-type D. melanogaster and the mutant strains of the fruit-fly stock, while the others have only been found in a small number of mutant strains, which confirms the low genetic diversity of the CS group.

For some of the Wolbachia genotypes, their effect on the fruit fly’s biological features has been described, e. g., clade V of the wMel genotype prevailing in the D. melanogaster population of the Palearctic (Bykov et al., 2019), and clade VI of the wMelCS genotype induce weak cytoplasmic incompatibility (Ilinsky, Zakharov, 2011; Ilinsky, 2013). Comparing the temperature survivability of flies (Versace et al., 2014; Mazzucco et al., 2020) has shown that those infected with clade V of the wMel genotype withstand cold temperatures better than those infected with clade VI of the wMelCS genotype and clades I, II, III of the wMel genotype. D. melanogaster also change their temperature preferences depending on the infection status and Wolbachia genotype (Arnold et al., 2019; Truitt et al., 2019). It has been demonstrated that wMelCS increases dopamine metabolism intensity unlike the wMel, wMel2 and wMel4 genotypes (Gruntenko et al., 2017). The female fruit flies infected with the wMel genotype are more productive than non-infected ones or those infected with the wMelCS genotype (Serga et al., 2014). The authors also note the wMelCS genotype is able to reduce the fruit fly’s fertility.

Many data have been accumulated to describe Wolbachia’s spread and variability in the wild D. melanogaster populations (Hoffmann et al., 1994, 1998; Riegler et al., 2005; Ilinsky, Zakharov, 2007a, b; Vesprool, Haddrill, 2011; Bykov et al., 2019), while the set of investigations studying the issue in the laboratory strains includes only two reports (Clark et al., 2005; Ilinsky et al., 2014). A study of the flies kept at Bloomington Drosophila Stock Center was carried out only to estimate the infection degree in wild-type strains and the strains containing different mutation groups and P-element (Clark et al., 2005). It demonstrated the differences in the number of infected lines for different groups of fruit flies, which were probably related to their breeding history.

The second study was carried out in the stock of Laboratory of Population Genetics of Institute of Cytology and Genetics, Siberian Branch of the Russian Academy of Sciences, and its objective was not only to detect Wolbachia infection frequency but also to estimate its genetic diversity in the mutant strains of the stock (Ilinsky et al., 2014). It has been found the line groups with different mutation differed both in terms of infection frequency and Wolbachia genotype composition. In some cases, it could be related to the breeding history, in particular, to using the specific infected balancing strains for maintaining certain mutations

When it comes to Wolbachia’s genetic diversity, the CS group is of particular interest for it is still unknown how these genotypes are maintained in D. melanogaster populations. Considering their low frequency, they should be eliminated in the populations either due to genetic drift or being replaced by the wMel genotype, but this is not what happens in reality (Riegler et al., 2005; Ilinsky, 2013; Bykov et al., 2019). It is likely that these genotypes are supported thorough selection. Recently, new data have been published concerning some phenotypic effects observed in this genotype group, e. g., the wMelPlus strain of wMelCS genotype increases the flies’ survival in presence of thermal stress. However, the mechanism of this phenomenon remains unknown (Burdina et al., 2021). Another strain (wMelPop) of the same genotype was detected when observing flies’ death due to rampant bacterial proliferation in the host’s cells (Min, Benzer, 1997; Woolfit et al., 2013).

Genetic differentiation and comparative analysis of Wolbachia isolates will make it possible to detect new effects and understand the mechanisms of host-symbiont interactions, which can later be used for practical applications, e. g., for the wMel and wMelCS genotypes, their ability to block mosquito-borne viral infections has been found. In other words, they prevent dengue fever, Zika virus infection and other viral infections when they are transmitted from the fruit fly to the mosquito (Schultz et al., 2017; Xue et al., 2018; Flores et al., 2020).

The aim of the present study was performing a search in the D. melanogaster strains of the laboratory stock of Institute of Cytology and Genetics of Siberian Branch of the Russian Academy of Sciences to detect the rare Wolbachia genotypes such as wMelCS and wMelCS2 as well as new genotypes. These strains can later be used to investigate the effect Wolbachia has on the biological features of D. melanogaster, in particular, to analyze its effect on the metabolism of infected fruit-fly strains, their fertility and thermal stress resistance. The results of our study will also complement to the early obtained data on Wolbachia diversity in natural and laboratory populations of D. melanogaster.

## Materials and methods

In the study, 555 strains of D. melanogaster from the laboratory stock of Institute of Cytology and Genetics of Siberian Branch of the Russian Academy of Sciences were used. The lines were bred from the natural populations collected in different regions of Russia, Ukraine and Kyrgyzstan from 1985 to 2016 as well as in Kenia in 2019 (Tables 1 and 2). For the DNA extraction, pools of five females were used. The flies were homogenized in STE buffer (100 mM NaCl, 10 mM Tris-Cl, pH 8.0, 1 mM EDTA, pH 8.0) and incubated during an hour at 56 °C. After the incubation, the samples were centrifuged at 13,000 RPM for 10 min for debris removal, and the supernatant was PCR assayed for 1) presence of Wolbachia (whole collection); 2) infection frequency and presence of rare Wolbachia genotypes (370 wild-type strains (see Table 1)); for the population represented with more than 10 strains, infection frequency was determined and 95 % confidence intervals (CI) was estimated using Clopper–Pearson method; 3) CS-genotype diversity (170 strains containing natural mutations (see Table 2)); 4) possible infection loss (15 strains of wild-type D. melanogaster from the Tomsk population (see Table 1) that had been earlier characterized in terms of their Wolbachia genotype and infection status (Bykov et al., 2019)).

**Table 1.**
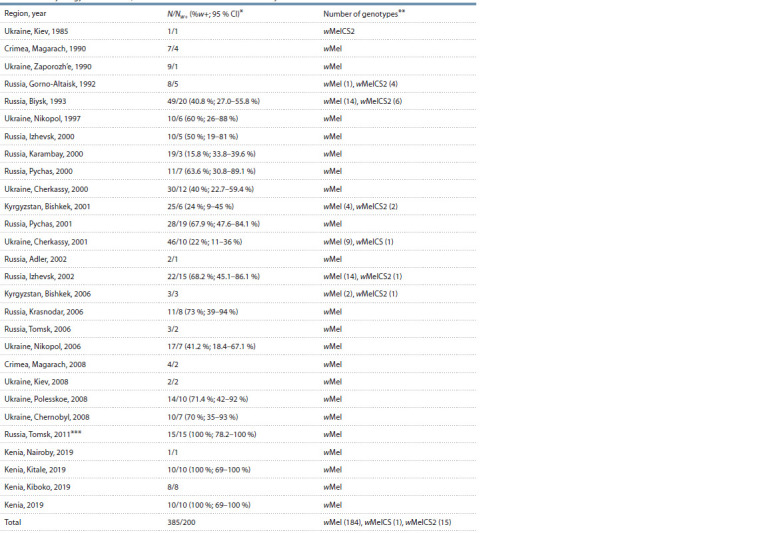
Wolbachia prevalence and genetic content in the wild-type D. melanogaster strains from the laboratory stock of Institute of Cytology and Genetics, Siberian Branch of the Russian Academy of Sciences * N – the number of assayed strains; Nw+ – the number of infected strains; %w+ – proportion of infected samples; 95 % confidence intervals were estimated using the Clopper–Pearson method for samples with N ≥ 10; ** the number are indicated in cases of several genotypes detected; *** the strains have been earlier characterized (Bykov et al., 2019).

**Table 2.**
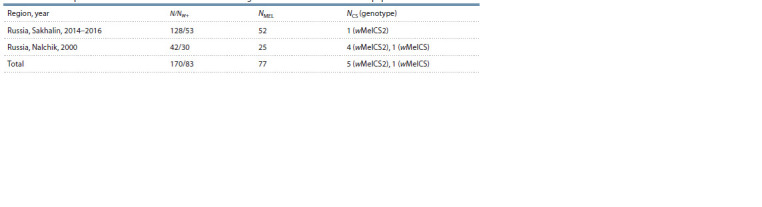
Wolbachia prevalence in the collection of mutant D. melanogaster strains derived from natural populations Notе. N – the number of assayed strains; Nw+ – the number of infected strains; NMEL, NCS – the number of the strains harboring Wolbachia of MEL and CS clades, respectively

The Wolbachia infection status and genotype were determined according M. Riegler et al. (2005) based on four markers such as insertion in WD_1310 and WD_0516 locus; the number of vntr 105 and vntr 141 minisatellite repeats. For the 170 mutant strains, Wolbachia presence was checked only for loci 1310 and 0516/7 to determine whether the bacteria belonged to the MEL or CS group. For the detected CS variants, additional assay for loci vntr 105 and vntr 141 was carried out to distinguish the wMelCS and wMelCS2 genotypes. These 170 strains were discarded from the analysis of the infection and genotype frequencies since they did not provide information on the symbiont’s prevalence in the population. Statistical analysis of the obtained data was performed using the Minitab 17.1.0 software (Minitab Inc., State College, PA, USA).

## Results

In the 555 strains of D. melanogaster assayed, the Wolbachia infection was detected in 51.9 % of wild-type (see Table 1) and 48.8 % of mutant (see Table 2) strains. In the assayed wild-type strains, the infection rate varied from 15.8 to 100 % (see Table 1), 52 % on average (95 % CI 46.8–57.0 %). The symbiont was detected in all population samples. Fifteen strains of the Tomsk population turned out to be infected with Wolbachia of expected genotype, i. e., no infection loss after 10 years of breeding was found.

Wolbachia diversity in the assayed wild-type D. melanogaster strains was represented by three genotypes wMel, wMelCS and wMelCS2. More than 90 % of infected strains carried the wMel genotype, that correlated with its dominance in natural populations worldwide (Riegler et al., 2005; Ilinsky, Zakharov, 2007a, b; Bykov et al., 2019). About 9 % of the infected strains obtained from the natural populations of Altai (Gorno-Altaisk, Biysk), Kyrgyzstan (Bishkek) and Udmurtia (Izhevsk) carried the wMelCS2 genotype. The only case of wMelCS was detected in a strain from a natural population of Ukraine. Rare CS-clade variants were also found in the mutant flies from the populations of Sakhalin and Nalchik (see Table 2). At the same time, the wMelCS genotype had never been found in Sakahlin earlier as well as wMelCS had never been detected in Nalchik.

## Discussion

In the present study, we carried out a search for the Wolbachia bacteria of wMelCS and wMelCS2 genotypes in the D. melanogaster strains collected from natural populations and maintained in laboratory stock for 3–36 years. These genetic variants of the symbiont are rare in natural populations but still can be widely spread worldwide (Riegler et al., 2005; Ilinsky, Zakharov, 2007a, b; Bykov et al., 2019). In the majority of cases in this study they were found in the strains from the regions where these genotypes had been registered earlier.

In Udmurtia (Izhevsk), the wMelCS2 genotype had never been registered in D. melanogaster, which was probably due to the small number of assayed strains (Ilinsky, Zakharov, 2007a). For the time being, this is the northernmost geographical location where this genotype has been registered (Bykov et al., 2019), but one has to keep in mind that we know quite a little about the northern populations of D. melanogaster and the boundaries of its spread can be much wider than the ones known to us today. At the same time, accidental delivery of D. melanogaster infected with this Wolbachia genotype together with products should not be excluded. So, later it may disappear from the local population due to the death of its hosts in the winter period. A similar case of accidental delivery was probably observed in the mutant strain from the Sakhalin Island. These flies had wMelCS-genotype Wolbachia that had never been registered in this territory.

Earlier, we characterize in detail the infection rate and genetic diversity of Wolbachia in D. melanogaster populations from Nalchik collected in 2010–2013, the single cases of wMelCS2 genotype were found (Bykov et al., 2014, 2019). Analysis of the mutant strains bred from the Nalchik population in 2000 demonstrated the presence of both wMelCS2 and wMelCS genotypes. The available data enable us to conclude that wMelCS2 is constantly supported in the populations of this region. The detected case of wMelCS genotype confirm our earlier assumption that this variant of bacteria can present in the fly populations of Nalchik (Bykov et al., 2014). The long-term presence of rare Wolbachia genotypes in D. melanogaster may be due to several reasons, e. g., the flies harboring the wMelCS and wMelCS2 genotypes may overwinter and produce new generations of infected insects (Kriesner et al., 2016; Bykov et al., 2019). Also, the symbiont itself may provide advantages for infected species (Hedges et al., 2008; Teixeira et al., 2008; Gruntenko et al., 2017) or induce the reproductive abnormalities that sustain infection in the population (Ilinsky, Zakharov, 2011; Ilinsky, 2013).

In the mutant laboratory strains of D. melanogaster, the wMelCS and wMelCS2 genotypes occur much more often, which is due to strains’ breeding history that involved using of the balancing strains infected with these Wolbachia genotypes (Ilinsky et al., 2014). Comparative analysis of Wolbachia genetic diversity in the natural, mutant and wild-type strains demonstrated the presence of statistically significant differences in genotype ratio between the stock’s wild-type strains and the natural populations (Fisher’s exact test, p = 7×10–5). The symbiont’s genetic composition in the mutant strains also differed significantly from that in the natural strains (Fisher’s exact test, p < 1×10–8 for both cases) (Table 3).

**Table 3.**
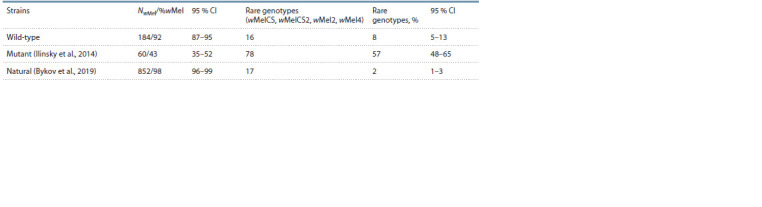
Comparison of Wolbachia’s genetic compositions in the wild-type, mutant and natural strains of D. melanogaster Notе. NwMel – the number of strains with Wolbachia of the wMel genotype; %wMel – percents of strains with Wolbachia of the wMel genotype.

In general, the Wolbachia prevalence in the stock’s cultures of D. melanogaster was comparable to those in the studies that had been published earlier, which confirms the symbionts is ubiquitous and its occurrence is of high frequency (Ilinsky, Zakharov, 2007a; Vespoor, Haddrill, 2011; Serga et al., 2014; Bykov et al., 2019). Detailed comparison of our data for wildtype strains to those for mutant strains and natural ones showed some differences in Wolbachia infection frequency, hence both mutant and wild-type strains were different from the natural ones (Fisher’s exact test, p = 0.043 and p < 1×10–8 for both cases). They differed from one another as well ( p = 0.0005) (Table 4).

**Table 4.**
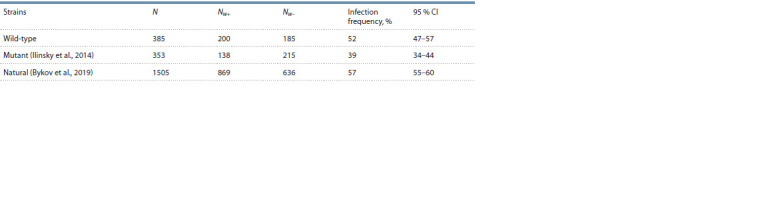
Wolbachia infection frequencies for the wild-type, mutant and natural strains of D. melanogaster Notе. N – the number of strains; Nw+, Nw– – the number of infected and uninfected strains, respectively.

A possible explanation of the differences in infection frequencies between natural and wild-type strains is to say some of the lines experienced infection loss after many generations. It is known that Wolbachia can eventually be lost in maternal lineage due to incomplete maternal transition, and in absence of any positive effect on its host can be completely eliminated from a population (Hoffmann et al., 1998; Ilinsky et al., 2014). Our analysis demonstrated that the symbiont preserved itself in the 15 lines of fruit flies from Tomsk, whose populations had been maintaining during 10 years. On the other hand, the mutant strains of D. melanogaster had demonstrated possible cases of infection loss (Ilinsky et al., 2014). In (Ilinsky, 2013), strain S400 infected with clade III of wMel genotype experienced infection loss (data not shown).

## Conclusion

The present study found two strains of D. melanogaster infected with the wMelCS genotype of Wolbachia, and 20 strains – with the wMelCS2 genotype. These strains will be further investigated to estimate the effect the symbiont has on the fruit fly’s biology. Our study has extended the boundaries of wMelCS2 spread, whose northernmost point now is Udmurtia (Izhevsk). Our results confirm Wolbachia can be sustained in laboratory strains, which does not exclude the likelihood of infection loss after long-term breeding. The symbiont’s infection frequency and genotypic composition are in general comparable to those estimated in natural populations and supplement the available data. When compared against those in the mutant strains, Wolbachia infection frequency and genotypic composition in the wild-type strains turned out to be closer to those observed in natural populations

## Conflict of interest

The authors declare no conflict of interest.
